# Anthranilate at the interface of tryptophan and specialized metabolite biosynthesis

**DOI:** 10.3389/fpls.2025.1625337

**Published:** 2025-07-08

**Authors:** Cynthia K. Holland, Aracely P. Watson, Ellia Chiang

**Affiliations:** Department of Biology, Williams College, Williamstown, MA, United States

**Keywords:** tryptophan, anthranilate, specialized metabolism, plant defense, volatiles

## Abstract

Plants synthesize a diverse array of specialized metabolites that contribute to plant development, growth, protection from biotic and abiotic stressors, and attracting pollinators and seed dispersers. Specialized metabolites are often derived from primary metabolites, such as amino acids, but also can be redirected from intermediates in primary metabolic pathways. In the L-tryptophan (Trp) biosynthetic pathway, the intermediate anthranilate is siphoned away to synthesize volatiles and specialized metabolites. Methyltransferases can produce the *O-*methyl ester of anthranilate, a grape aroma volatile produced in species such as grapevine, strawberry, citrus, maize, and soybean. *O*-Methyl anthranilate serves context-dependent roles in attracting insects and deterring herbivores. Methylation at the amine generates *N*-methyl anthranilate, a precursor for *N*-methyl anthranilate esters in citrus and antimicrobial avenacins in black oat. This Mini Review explores the regulation of anthranilate within the context of the Trp pathway and its contributions to the biosynthesis of anthranilate-containing volatiles and specialized metabolites. Also highlighted are the roles of anthranilates in plant defensive metabolism and the substrate specificity of anthranilate-using enzymes, as well as unanswered questions about the synthesis, transport, and physiological role of anthranilates.

## 1 Introduction

Plants synthesize a wide array of small molecules that aid in chemical defense against pathogenic microorganisms and herbivores (Maeda and Fernie, 2021). These molecules are typically secondary metabolites that are built from primary metabolites, such as amino acids, fatty acids, isoprene, nucleic acids, or intermediates in primary biosynthetic pathways (Jacobowitz and Weng, 2020). Some of the secondary metabolites produced in leaves, flowers, and roots are emitted as volatiles. These volatiles can be synthesized from five-carbon isoprene units, fatty acids, aromatic rings, or amino acids (Dudareva et al., 2013). Volatiles and secondary metabolites serve a number of roles in plants, namely in mediating interactions between plants and herbivores, pollinators, seed dispersers, and microorganisms (Schuman, 2023).

**Figure 1:**
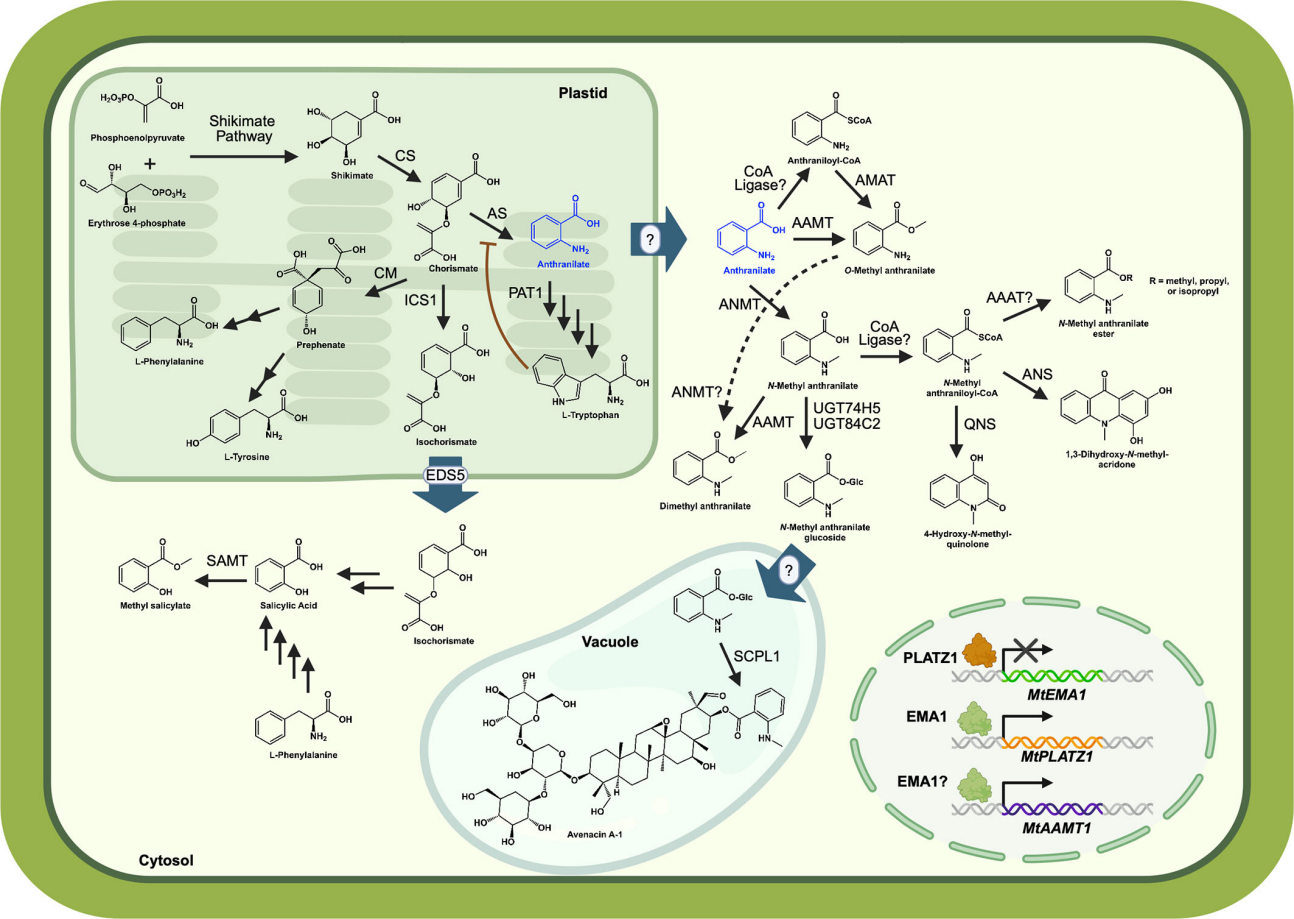
Anthranilate branchpoint in plants. Anthranilate (blue) is synthesized in the plastid as part of the tryptophan biosynthesis pathway, and is likely transported to the cytosol where it serves as a precursor to volatiles and specialized metabolites. Trp inhibits some anthranilate synthases (AS), denoted by the red line. The transport protein (blue arrow) is known for isochorismate, but not for anthranilate. Expression of the *Medicago truncatula AAMT1* (*anthranilate methyltransferase*) is mediated by EMA1 (emission of methyl anthranilate 1), while PLATZ1 (plant AT-rich sequence and zinc-binding 1) represses the expression of *EMA1*. CS, chorismate synthase; PAT1, anthranilate phosphoribosyltransferase; CM, chorismate mutase; ICS1, isochorismate synthase 1; EDS5, enhanced disease susceptibility 5; SAMT, salicylic acid methyltransferase; ANMT, anthranilate *N-*methyltransferase; AMAT, anthraniloyl-CoA:methanol acyltransferase; AAAT, anthraniloyl-CoA:alcohol acyltransferase; UGT, UDP-dependent glycosyltransferase; QNS, quinolone synthase; ANS, acridone synthase; SCPL1, serine carboxypeptidase-like acyl transferase. Salicylic acid biosynthesis (Lefevere et al., 2020) and avenacin biosynthesis and localization (Orme et al., 2019) are described in more detail in recent publications. Figure was prepared using BioRender.

All plants synthesize anthranilate as an intermediate in the L-tryptophan (Trp) biosynthetic pathway, and anthranilate and its derivatives (i.e., anthranilates) have multiple fates in plants (**Figure 1**). Unlike mammals, plants have the enzymatic machinery to synthesize all 20 amino acids, and Trp is an aromatic amino acid that is essential for the synthesis of proteins, the growth hormone auxin, niacin (vitamin B3), and specialized metabolites like indole glucosinolates in the mustard family (*Brassicaceae*) and the monoterpene indole alkaloids vinblastine and vincristine in Madagascar periwinkle (*Catharanthus roseus*) (Radwanski and Last, 1995; Sønderby et al., 2010; Maeda and Dudareva, 2012; Caputi et al., 2018; Morffy and Strader, 2020). Despite the importance of Trp biosynthesis in both primary and secondary metabolism, the Trp pathway enzyme that acts on anthranilate, anthranilate phosphoribosyltransferase, was only recently biochemically characterized (Li et al., 2023).

In at least 19 plant families, anthranilate is methylated to form a volatile ester that is responsible for grape aroma (Knudsen et al., 2006). Plant volatiles like *O-*methyl anthranilate (*O-*MeAA) are released following mechanical damage and function in mediating defense against predators. In strawberries (*Frageria* spp.), *O*-MeAA attracts *Drosophila suzukii* pests that preferentially oviposit on high concentrations of *O*-MeAA; however, *O*-MeAA has a concentration-dependent effect on embryo lethality (Bracker et al., 2020). In maize, an *O*-MeAA-containing blend of volatiles is emitted upon insect-induced tissue damage, which attracts wasps that parasitize the insect (Turlings et al., 1990; von Merey et al., 2013). In plants that don’t synthesize *O-*MeAA, exogenous application of the volatile on crops is effective in attracting natural enemies of insect herbivores to prevent further damage (Simpson et al., 2011). *O-*MeAA also functions as an effective bird repellant (Avery et al., 1995). Anthranilate methyltransferases responsible for generating *O-*MeAA were recently identified in *Vitis* spp. (grapevine), and biochemical investigations have enabled the identification of key residues that confer anthranilate activity in these enzymes (**Figure 1**) (Fallon et al., 2024).

Plants such as black oat (*Avena strigosa*) and members of the Rutaceae family, including Mexican orange blossom (*Choisya ternata* Kunth) and common rue (*Ruta graveolens*), methylate the amine of anthranilate (**Figure 1**) (Rohde et al., 2008; Mugford et al., 2013). In black oat, *N-*methyl anthranilate (*N*-MeAA) is incorporated into the chemical structure of avenacins, which are anti-microbial small molecules that are synthesized in root epidermal cells (Papadopoulou et al., 1999). In Rutaceae species, *O-*methyl esters of *N*-MeAA, such as dimethyl anthranilate (DiMeAA), exhibit pain-relieving activity in mice (Pinheiro et al., 2014); however, their role in mediating plant responses to biotic stress remains unclear.

This Mini-Review summarizes recent investigations of anthranilate-using enzymes in primary and secondary metabolism, as well as the substrate specificity and regulation of these enzymes. Additionally, this review highlights the need for future studies to increase our understanding of the regulation, localization, and transport of anthranilates, as well as their contributions to plant physiology.

## 2 Anthranilate is an intermediate in tryptophan biosynthesis

Until recently, much of what is known about Trp biosynthesis and regulation had been inferred from microbial investigations of these pathways (Maeda and Dudareva, 2012). Trp biosynthesis occurs in the plastid and emanates from the product of the shikimate pathway. The shikimate pathway starts by combining erythrose 4-phosphate (E4P) from the light-independent reactions of photosynthesis with phosphoenolpyruvate (PEP) from glycolysis. This seven-step pathway gives rise to chorismate, which serves as a precursor to the aromatic amino acids phenylalanine (Phe), tyrosine (Tyr), and Trp and to the plant hormone salicylic acid (SA) (**Figure 1**) (Niyogi and Fink, 1992; Westfall et al., 2014; Kroll et al., 2017; Spoel and Dong, 2024).

Anthranilate synthase (AS) catalyzes the first, and committed, step in Trp biosynthesis to convert chorismate to anthranilate and pyruvate (**Figure 1**) (Niyogi and Fink, 1992; Niyogi et al., 1993; Romero et al., 1995; Tozawa et al., 2001; Inaba et al., 2007). The amine that is added to chorismate has two potential sources: free ammonia (NH_3_) or the side chain amine of glutamine (Gln), depending on the AS subunits used to catalyze the reaction. Free ammonia is used when ASα acts as a monofunctional protein, while the ASα/β heterodimer uses Gln as the amine donor (Niyogi and Fink, 1992; Niyogi et al., 1993). AS is feedback regulated through allosteric inhibition by Trp, although ASαs that are feedback-insensitive to Trp have been identified in rice, common rue, and tobacco (Bohlmann et al., 1996; Song et al., 1998; Tozawa et al., 2001). Investigations of the source of this Trp insensitivity have identified several residues responsible for feedback inhibition in both plants and bacteria (Caligiuri and Bauerle, 1991; Bernasconi et al., 1994; Kreps et al., 1996). Transcriptional regulation of AS isozymes provides an additional layer of control. *Arabidopsis* contains two ASαs, and evidence suggests that ASα2 is expressed at low, constitutive levels, while ASα1 is induced during wounding and bacterial infiltration (Niyogi and Fink, 1992).

In the second step of the pathway, anthranilate phosphoribosyltransferase (PAT1) transfers a phosphoribosyl sugar onto anthranilate, forming 5-phosphoribosylanthranilate (Last and Fink, 1988; Rose et al., 1992; Radwanski and Last, 1995). The *PAT1* gene was the first Trp pathway enzyme discovered in plants (Last and Fink, 1988). EMS-mutagenized *Arabidopsis thaliana* seedlings were grown on Trp and 5-methyl anthranilate (5-MA), which is converted to 5-methyl-Trp in plants that have a fully functional Trp pathway downstream of anthranilate. Mutant seedlings that grew on Trp and were resistant to 5-MA were named *trp1-1* and were found to lack phosphoribosyl transferase activity. Because *PAT1* is a single-copy gene in *Arabidopsis*, *trp1-1* plants require Trp in the growth media. The *trp1-1* mutants also exhibit blue fluorescence under UV light. The *trp1* mutant plants that were supplemented with Trp appeared similar to wild type (WT) initially, but after 3-4 weeks, the plants were smaller than WT, a phenotype which could not be rescued with exogenous Trp (Last and Fink, 1988). The *trp1-1* plants were bushy, had small and crinkled rosette leaves, and are virtually sterile. Another mutant allele, *trp1-100*, grew and developed normally without exogenous Trp, yet maintained the blue fluorescence phenotype and exhibited normal fertility (Rose et al., 1992). The source of this blue fluorescence was fortuitously discovered as a result of anthranilate β-glucoside accumulation in the *trp1* mutants via glucosylation by UGT74F2, and the fluorescence phenotype was rescued in *trp1 ugt74f2* double mutant plants (Quiel and Bender, 2003).

PAT1 remained biochemically uncharacterized until recently (Li et al., 2023). By comparing structural models, the putative active site residues of 82 PAT1 enzymes were compared, and the six enzymes with the most variability in the active site were characterized using steady-state kinetics. PAT1s from *A. thaliana* and *Citrus sinensis* (sweet orange) exhibited the highest catalytic efficiencies with anthranilate, followed by the PAT1s from *Physcomitrium patens* (spreading earth-moss), *Juglans regia* (English walnut), and *Pistacia vera* (pistachio). The PAT1 from *Selaginella moellendorffii* (spike moss) exhibited the lowest catalytic efficiency, with a more than 14-fold reduction relative to that of *A. thaliana*. The enzymes followed substrate inhibition kinetics with high concentrations of anthranilate leading to reduced enzymatic activity.

With the exception of the *C. sinensis* PAT1, these enzymes could also act on 3-hydroxyanthranilate (3-HAA), a Trp catabolism intermediate in mammals that has been reported in maize (Tarr and Arditti, 1982; Li et al., 2023). Comparative active site analysis between *C. sinensis* PAT1 and the *A. thaliana* PAT1, which had the highest catalytic efficiency with 3-HAA, identified Asn215 in AtPAT1 as a residue that confers 3-HAA activity (Li et al., 2023). While PAT1 is not known to have an allosteric site, Tyr inhibited the *P. patens* PAT1 with an IC_50_ value of 1180 μM, while Phe inhibited the *S. moellendorfii* PAT1 with an IC_50_ value of 1980 μM. The activity of the four other PAT1s were not modulated by aromatic amino acids.

Notably, the *C. sinensis* PAT1, which has a high catalytic efficiency with anthranilate, is insensitive to modulation by aromatic amino acids (Li et al., 2023). This functional data may explain how Rutaceae species balance anthranilate allocation to Trp biosynthesis with competing biosynthetic pathways for producing *O*-MeAA, *N*-MeAA esters, acridone alkaloids, and quinolones. Furthermore, enzyme-level regulation via substrate inhibition may balance flux through primary and specialized metabolism to ensure sufficient Trp is produced to promote plant growth and development.

## 3 Biosynthesis of anthranilate methyl esters

*O-*MeAA biosynthesis has only been investigated in a handful of crop plants, including soybean (*Glycine max*) (Lin et al., 2013), maize (*Zea mays*) (Köllner et al., 2010), barrel clover (*Medigaco truncatula*) (Pollier et al., 2019), strawberries (*Frageria* spp.) (Pillet et al., 2017), grapes (*Vitis* spp.) (Wang and De Luca, 2005; Fallon et al., 2024), and sweet orange (*C. sinensis*) (Huang et al., 2016). There are two biosynthetic routes for *O-*MeAA: one uses a one-step SAM-dependent anthranilate *O-*methyltransferase (AAMT) and the other uses a two-step pathway. In the two-step pathway, Coenzyme-A (CoA) is first added to the carboxylate of anthranilate by a ligase that remains to be identified (**Figure 1**). This anthraniloyl-CoA is then combined with methanol using an anthraniloyl-CoA:methanol acyltransferase (AMAT) to produce *O-*MeAA (Wang and De Luca, 2005; Yang et al., 2020).

Although *O-*MeAA imparts the classic grape aroma, the complete biosynthetic pathway for *O-*MeAA in grapes remained enigmatic until recently. While one-step AAMTs had been characterized as the mechanism for *O-*MeAA biosynthesis in other plants, grapes were thought to primarily rely upon the two-step pathway until the recent identification of two one-step AAMTs in *Vitis* (Fallon et al., 2024). In *C. sinensis*, a SA methyltransferase (SAMT) has activity with anthranilate, and comparisons between the newly identified grape AAMTs and the *Citrus* SAMT led to the identification of three residues (Gln263, Cys319, and Ala324) that confer anthranilate activity. Similar comparisons using the maize AAMT1, which is highly specific for anthranilate, identified three residues (Cys165, Tyr246, and Leu329) that increase activity with SA (Köllner et al., 2010; Fallon et al., 2024). Interestingly, one of the grape AAMTs and the strawberry AAMT have shared ancestry with jasmonate methyltransferases (Pillet et al., 2017; Fallon et al., 2024).

The regulation of *AAMT* expression has been shown to be induced by methyl jasmonate in maize and in the legume *Medicago truncatula* (Köllner et al., 2010; Pollier et al., 2019). In *M. truncatula*, an R2-R3 MYB transcription factor Emission of Methyl Anthranilate 1 (EMA1) was found to promote *AAMT* expression in hairy roots (Pollier et al., 2019). Treating seedlings with *O-*MeAA led to a several thousand-fold increase in *EMA1* transcript levels after 24 hours. Additionally, the transcriptional repressor plant AT-rich sequence and zinc-binding 1 (PLATZ1) regulated *EMA1* expression and thus volatile biosynthesis (**Figure 1**). Future experiments are needed to identify additional transcriptional regulators of *AAMT* gene expression in plants and to gain a more complete understanding of the regulatory network underlying *O*-MeAA biosynthesis across *O*-MeAA-producing species.

## 4 *N*-methyl anthranilate-containing specialized metabolites

In addition to methylation at the carboxyl group, anthranilate can also be methylated on the amine by an anthranilate *N*-methyltransferase (ANMT). ANMTs has been identified in black oat (*A. strigosa*) and common rue (*R. graveolens*) (Rohde et al., 2008; Mugford et al., 2013). Members of the Rutaceae family are notable for their diversity of *N*-MeAA metabolites, where *N*-MeAA serves as a precursor for acridone alkaloids and anti-malarial quinolone alkaloids (Rohde et al., 2008; Mori et al., 2013). A type III polyketide synthase, acridone synthase, condenses *N*-methyl anthraniloyl-CoA with three malonyl-CoA units to form 1,3-dihydroxy-*N*-methylacridone (Maier et al., 1993), while the condensation of *N*-methyl anthraniloyl-CoA with one unit of malonyl-CoA by quinoline synthase gives rise to 4-hydroxy-*N*-methyl-quinolone (Resmi et al., 2013). Acridone, acridine, and their derivatives are toxic to human cell lines and have broad biological activity against bacteria, viruses, and parasites and inhibit acetylcholinesterase, suggesting that they may serve a defensive role in plants (Kelly et al., 2009; Gensicka-Kowalewska et al., 2017). Quinolones are also synthesized by plants in the Rubiaceae family (Michael, 2003), and quinine from *Cinchona* bark has been used to treat malaria parasite (*Plasmodium* spp.) infections in humans (Gachelin et al., 2017).

Various *N-*MeAA esters including DiMeAA, propyl-*N-*MeAA, and isopropyl-*N-*MeAA, have been identified in Mexican orange blossom leaves (*Choisya ternata* Kunth) (Pinheiro et al., 2015). These esters are thought to be synthesized using an acyl-CoA:alchocol acyltransferase, homologous to the AMAT in grapes (Wang and De Luca, 2005); however, a CoA ligase that acts on anthranilate or *N*-MeAA remains to be identified in plants (**Figure 1**). These anthranilates exhibit dose-dependent anti-nociceptive effects, as well as anti-anxiety and anti-depressive activity in mice (Radulovic et al., 2013; Pinheiro et al., 2015). However, their role in plant metabolism and plant responses to biotic factors has yet to be investigated. DiMeAA was also identified as a quorum sensing inhibitor with antibiofilm activity against the foodborne pathogen *Pseudomonas aeruginosa* (Ma et al., 2021), suggesting that it may be involved in plant-microbe interactions.

Oats release avenacins, which are anti-microbial glycosylated triterpenes that are acylated at C-21 with either *N*-MeAA (A-1 and B-1) or benzoic acid (A-2 and B-2), from their roots to protect against pathogens in the soil (**Figure 1**) (Mugford et al., 2013; Owatworakit et al., 2013). The connections between the avenacins and their anti-microbial properties were made by screening oat genotypes for susceptibility to *Gaeumannomyces graminis* var. *tritici*, a fungus that causes take-all disease in oats, which unveiled that the susceptible *A. longiglumis* did not produce detectable levels of avenacins (Osbourn et al., 1994). Follow-up studies identified the 12-gene avenacin biosynthetic gene cluster (Li et al., 2021), which included the glycosyltransferase UGT74H5 (SAD10) that activates *N*-MeAA for addition onto avenacin A-1 and B-1 (Mugford et al., 2009; Owatworakit et al., 2013). *N*-MeAA-glucoside is imported into the vacuole, which is where it is conjugated onto avenacins via a SAD7 serine carboxypeptidase-like acyl transferase (SCPL1) (Orme et al., 2019). Recently, a nonclustered gene that encodes a glycosyltransferase, UGT84C2 (SAD4), was found that also activates *N*-MeAA via glucosylation for SAD7-mediated transfer onto A-1 and B-1 avenacins (Qiao et al., 2025). Aside from oats and Rutaceae species, *N*-methylated anthranilates have not been studied. It is likely that chemical profiling of more plants in the future will turn up additional plants that synthesize anthranilate-derived specialized metabolites.

## 5 Discussion

While all plants synthesize anthranilate as a Trp pathway intermediate, specialized metabolites and volatiles containing anthranilate represent an under-explored area of plant metabolism. There is still much to learn regarding how plants regulate Trp biosynthesis via anthranilate synthase and the extent to which other amino acids or plant metabolites regulate PAT1. Anthranilate is synthesized in the plastid where Trp biosynthesis occurs, but anthranilate-using enzymes in specialized metabolism, such as ANMTs, localize to the cytosol or are predicted to be cytosolic based on the absence of a signal peptide (Mugford et al., 2013; Fallon et al., 2024) (**Figure 1**). The plastid-to-cytosol transport mechanism for anthranilate remains to be investigated, as does the mechanisms regulating subcellular partitioning of anthranilate.

Recent advances in identifying anthranilate-using enzymes in specialized metabolism increases our understanding of the molecular basis of anthranilate recognition by enzymes such as glycosyltransferases and methyltransferases (Owatworakit et al., 2013; Fallon et al., 2024). Single amino acid changes to active site residues in the maize AAMT1 were sufficient to increase SA production 8-fold when heterologously expressed in *E. coli* cultures, which has enabled the identification of amino acids that confer activity with anthranilate versus SA (Fallon et al., 2024). Similarly, single mutants of the *C. sinensis* SAMT led to increased activity with anthranilate. Comparisons between the *Ruta graveolens* ANMT and a caffeate *O*-methyltransferase identified the molecular basis of chemoselectivity for *N*- versus *O*- methylation (Jockmann et al., 2025).

While *O-*MeAA mediates plant-insect interactions, there appears to be context dependence to these interactions. For example, maize induces *O*-MeAA biosynthesis upon insect herbivory and induces *AAMT1* expression (Köllner et al., 2010), while *C. sinensis* plants shut off *O*-MeAA release when they are attacked by the psyllid-vectored bacterial pathogen *Candidatus* Liberibacter asiaticus (CLas) that causes citrus greening (huanglongbing) (Mann et al., 2012). Aside from the EMA1 transcriptional regulator that was identified in *Medicago truncatula*, additional research is needed to identify the genetic regulators of *O*-MeAA biosynthesis in plants in order to better understand the species-specific differences underlying *O*-MeAA emission. Furthermore, *O*-MeAA is emitted as a part of a volatile blend, and it is likely the combination of volatiles that aid in insect attraction or deterrence (von Merey et al., 2013; Zhou and Jander, 2021).

Anthranilate-containing specialized metabolites and volatiles are likely more widespread across Viridiplantae and have been undersampled in plants, especially considering how nearly all angiosperm orders have a SAMT that could conceivably also act on anthranilate (Dubs et al., 2022; Fallon et al., 2024). Except for the avenacins in *A. strigosa* and *O*-MeAA in *M. truncatula*, anthranilates have escaped investigation in below-ground tissues (Pollier et al., 2019; Li et al., 2021). Understanding the tissue-level and developmental patterns of the synthesis of anthranilates may aid in gaining a more comprehensive understanding in their roles in plant physiology and biotic interactions.

In summary, anthranilates are an under-investigated area of plant metabolism, and many open questions remain regarding: the identification of biosynthetic genes; the transcriptional and enzyme-level regulation of anthranilate specialized metabolic pathways; the transport of anthranilate between subcellular compartments; the diversity of plants that synthesize anthranilates; and the role of anthranilates in plant physiology.
